# The St. John Ambulance Association

**Published:** 1897-08-07

**Authors:** 


### THE ST. JOHN AMBULANCE ASSOCIATION.

By a Practising Physician.

The large, important nature of the work of this association may be gathered from the fact that the grand total of certificates in "First Aid," and "Nursing," issued up to the present date is nearly 370,000, whilst 43,800 persons who have completed a three years' course in these subjects have been granted medallions. The Brigade, too, now numbers some 7,000 officers and men, whose services on public occasions, such as the recent Jubilee celebrations, have proved invaluable. The Invalid Transport Corps has during the past year removed 650 patients, and since the opening of the permanent station at St. Paul's upwards of 1,700 cases have received treatment. The Association, however, is still bent upon enlarging its sphere of work and usefulness, and purposes during the coming year to conduct examinations in "Cottage Nursing," and in "Hygiene." In order that the newly-created body of cottage nurses, who will be trained under what is known as the "Holt-Ockley system," may not unfairly compete with the duly trained, professional nurse, it is proposed that each certificate granted shall have specifically detailed on its back a list of the subjects in which the candidate has been examined and passed. The necessity for some such distinction is obvious, but the method proposed seems hardly sufficient to secure this end.

With regard to the teaching of hygiene, departing altogether from its old-established custom of selecting its lecturers solely from the medical staff, the association now proposes that this particular subject shall be taught not only by medical men but by holders of honours certificates from her Majesty's Science and Art Department, graduates of science of any recognised university, or by persons of either sex who have passed a special examination on a more advanced and comprehensive syllabus for a "teacher's certificate" before an examination board appointed by the St. John Ambulance Association. It is to be feared that this change will not only tend to minimise the importance of the study of hygiene, but will eventually lower the value of the certificates granted by the association, for this value has depended to a great extent upon the fact of the lectures and examinations being conducted by qualified medical men. Neither the South Kensington certificate nor a degree in science qualifies a man in any way to become a teacher of hygiene. What the value of the association's "teacher's certificate" may be it is impossible to say, as details of the syllabus and nature of the examination are not yet disclosed. It is to be hoped that whilst every encouragement is given to the teaching of sanitation, some considerable alterations will be made in the proposed scheme before its final adoption.

